# A Preliminary Conceptual Framework of Clinical Documentation Burden: Exploratory Factor Analysis Investigating Usability, Effort, and Perceived Burden among Health Care Providers

**DOI:** 10.1055/a-2751-1896

**Published:** 2025-12-12

**Authors:** Rhiannon Doherty, Abby Swanson Kazley, Eva Karp, Jennifer Ferrand

**Affiliations:** 1 College of Health Professions – Medical University of South Carolina, Charleston, South Carolina, United States; 2 Department of Well-Being - Hartford Healthcare, Hartford, Connecticut, United States

**Keywords:** electronic health records (MeSH), cognitive load (MeSH), burnout, professional (MeSH), usability (MeSH), health care providers (MeSH), clinical documentation burden, psychological factors, EHR optimization

## Abstract

**Background:**

For every 30 minutes a provider spends seeing a patient, they spend 36 minutes charting in the electronic health record (EHR). Clinical documentation burden in U.S. health care is driven by increasing administrative tasks associated with EHRs, regulatory demands, and workflow inefficiencies. This burden contributes to increased cognitive load, fragmented care, and staff burnout. No comprehensive conceptual framework guides researchers addressing these challenges.

**Objectives:**

This study aimed to develop a conceptual framework clarifying the interplay between psychological factors, technology, and documentation attributes—usability, effort, and perceived burden—among health care providers.

**Methods:**

Data were collected from a cross-sectional survey using a convenience sample of hospital- and ambulatory-based physicians, advanced practice registered nurses, and physician assistants. A newly constructed questionnaire was used, incorporating elements from well-established instruments. Descriptive and exploratory factor analysis was performed to identify significant findings and develop the preliminary Clinical Documentation Burden Framework.

**Results:**

The analysis revealed three main factors underpinning clinical documentation burden: Poor usability, perceived task value, and excessive mental exertion. These factors were significantly correlated with professional dissonance (PD) and burnout, underscoring the complex interplay between time requirements, design challenges, task engagement, and cognitive load. The resulting conceptual framework highlights the importance of aligning documentation tasks with provider values to mitigate burden.

**Conclusion:**

The study offers new insights into the complex phenomenon of documentation burden affecting health care providers by incorporating key psychological factors. This conceptual framework provides a preliminary foundation for understanding this multifaceted problem. Like prior burnout research, conceptual clarity is key to creating shared definitions and a dedicated measurement instrument to support effective interventions. Given that the sample was predominantly advanced practice providers with underpowered subgroup comparisons, the framework should be interpreted as preliminary. This new appreciation of the dimensionality of documentation burden expands the potential levers available to alleviate operational strain and reduce PD and burnout.

## Background and Significance

As electronic health records (EHRs) become increasingly prevalent in health care delivery, documentation requirements and administrative demands have expanded, resulting in an increased documentation and information burden for clinical care providers. 1 2 3 4 5 6 7 An article in JAMA reported that physicians spend approximately 36.2 minutes documenting in the EHR for every 30-minute office visit. 8 Clinical documentation burden is defined as “the stress imposed by the excessive work required to generate clinical records of health care-related interactions, occurring as a result of the imbalance between the usability [of] and satisfaction [with] systems of health records keeping with clinical and regulatory demands of entering and consuming health records data.” 9 Excessive documentation is linked to an increase in medical errors, patient safety threats, job attrition, and burnout. 10 11

Clinical documentation burden in the U.S. health care system is driven by increasing administrative tasks associated with EHRs, regulatory and reimbursement demands, and workflow inefficiencies. 12 13 14 15 16 Significant usability issues in EHR design, including ineffective support and interface design problems, have increased cognitive burden, staff burnout, and fragmented care. 11 17 18 19 20 21 Lack of standardization and poor interoperability among EHR systems further increase the documentation workload and after-hours documentation time. 11 14 15 22 23 This burden is compounded by the redistribution of administrative tasks to clinicians, which further heightens cognitive load and reduces patient interaction. 22 24

Concerns about excessive documentation length and shifting toward desktop medicine, which detract from direct patient care, are prevalent. 25 26 27 Health care professionals often view administrative tasks as less meaningful, which contributes to dissatisfaction and burnout and negatively impacts patient–physician interactions. 2 5 28 29 30 Research indicates that reducing physicians' documentation time and effort can enhance productivity and improve patient-centered care. 31 Furthermore, when providers view documentation tasks as meaningful, they are less likely to perceive them as burdensome, thereby reducing their risk of burnout. 32 33 34 35 36 37 38

Despite the importance of addressing the clinical documentation burden, there is no consensus on a precise definition or framework for measuring it, 10 39 nor sufficient evidence to validate the measures. 40 The American Nursing Informatics Association outlined six domains of documentation burden, and initiatives like the 25 × 5 Symposium and the National Burden Reduction Collaborative have prioritized defining and measuring this burden to develop effective interventions. 9 14 41 Lack of standardization complicates comparisons and generalizations, highlighting the need for further research to develop a shared and more comprehensive framework that facilitates effective measurement and intervention strategies. 42 Notably, most burden research has focused on physicians, with far less attention to advanced practice providers (APPs), despite their overlapping diagnostic and treatment responsibilities.

Recent research on clinical documentation burden has focused on intervention development or describing the workload induced by documentation tasks while exploring the compounding effects of usability and other mandated requirements. 25 40 42 While the interventions described often improved EHR satisfaction and reduced documentation time, they did not consistently reduce burnout, highlighting the need for further investigation. 43 44 The fact that burden research lacks an appropriate conceptual framework is a barrier to synthesis and the development of cumulative science. 45 Work is underway to galvanize the community around a standard definition of “excessive DocBurden,” but the effort to map causal pathways remains uncharted. 25

Researchers, including the authors of this paper, currently borrow well-built tools (e.g., System Usability Scale [SUS]) because they measure aspects of the burden experience. However, without being designed to capture the unique attributes of the documentation burden, this results in construct undercoverage and an inconsistent factor structure. In the past, early burnout studies also suffered from ad hoc measures and heterogeneous interventions. Progress accelerated only after researchers identified the concept definition and dimensionality, developed explanatory models, and finally, standardized instruments, such as the Maslach Burnout Inventory. 37 A purpose-built framework is a prerequisite for a psychometrically sound, context-specific instrument, which allows researchers to replicate burnout's trajectory toward robust, actionable knowledge.

Study findings reveal that developing a conceptual framework to describe this phenomenon requires addressing the gap in understanding the relationship between documentation workload and perceived burden by incorporating clinical documentation attributes and the interplay of key psychological factors. A consistent pattern of findings allows us to postulate a specific concept of documentation burden as the cumulative effect of task demands, mediated by usability and moderated by personal and environmental resources, leading to outcomes such as perceived burden, professional dissonance (PD), and burnout. There are several assumptions: Specifically, the multifaceted nature of burden, the importance of individual perception and experience, the presence of psychological factors, and the influence of systemic factors.

Understanding how providers view the importance of documentation can affect their motivation and engagement with these tasks. 35 36 37 38 When tasks align with personal and professional values, perceived stress decreases, but many providers feel that documentation tasks are unnecessary and detract from patient care. 12 46 PD arises when there is a disconnect between providers' values and their work environment, leading to moral distress, reduced job satisfaction, and increased burnout, particularly when tasks are perceived as unnecessary or misaligned with their qualifications. 26 47 48 By understanding and integrating perceived task value into our understanding of documentation burden, organizations will be better equipped to tailor interventions to improve efficiency and satisfaction. This approach will inform EHR system design and policy decisions around the prioritization of documentation practices that maximize perceived value and minimize unnecessary tasks, leading to a more supportive and efficient health care environment.

## Objectives

The objective of this work was to develop a preliminary conceptual framework that clarifies the interplay between psychological factors, technology, and clinical documentation attributes as they apply to inpatient and outpatient direct-care practice among providers, including both physicians and APPs. To accomplish this, we

1. Used a national provider survey to explore the relationship between documentation burden and known contributing factors (e.g., poor usability and cognitive effort, and the suspected factor of perceived task value).
2. Integrated findings of survey data into a preliminary conceptual framework. We performed an exploratory factor analysis (EFA) on the data to identify significant findings, then adapted established theoretical models to the clinical documentation workflow and overlaid the identified burden items and factors to develop the Clinical Documentation Burden Framework.

## Materials and Methods

We used a cross-sectional survey to collect data from a convenience sample of diverse physicians and APPs. This study was reviewed by the Medical University of South Carolina's Institutional Review Board (IRB) and approved with Exemption Category 2. The primary analytical method was quantitative analysis of self-reported questionnaire data. Qualitatively analyzed open-ended questions supplemented the primary analysis and evaluated contextual alignment between respondents' narrative statements and their scored measure, utilizing a triangulation design. 49 By applying EFA, we uncovered underlying patterns explaining the variance in responses across items, ideally revealing latent dimensions and relationships related to documentation task burden and the role of perceived task value for health care providers. 50 The results of this interpretation form the basis of theoretical insights, leading to the development of a visual conceptual framework.

### Sampling

The sample consisted of physicians and APPs who clinically work at least part-time in inpatient or outpatient care across various specialties, whose daily work includes clinical documentation. The inclusion criteria were being actively engaged in clinical practice, able to provide informed consent, and having English fluency. Exclusion criteria were not practicing in a clinical setting (e.g., retired or having an administrative-only role). We recruited participants through three methods: (1) Opt-in participation from informatics-focused professional listservs, (2) purposive and snowball sampling via email invitations, and (3) targeted social media posts. Respondents were primarily advanced practice registered nurses (APRNs) and physician assistants (PAs); the smaller physician subsample precluded adequately powered subgroup comparisons, which we therefore report descriptively rather than inferentially. We utilized Research Electronic Data Capture (REDCap) as the method of data capture. The survey link opened onto a landing page with an IRB-approved information sheet, followed by a series of screening questions to confirm that respondents met the inclusion criteria.

### Data Collection

After confirming the absence of a published questionnaire that met the study's needs, we constructed a new questionnaire ( Supplementary Appendix A [Understanding Clinical Documentation Burden, available in the online version only]) based on a scoping review of the literature, which resulted in the identification of three overarching dimensions: Time investment, task evaluation, and perceived burden. 51 We operationalized these concepts as usability, effort, and value (see Supplementary Appendix B [available in the online version only]). We constructed our items from extractions of the System Usability Questionnaire, 52 the NASA Task Load Index (NASA-TLX), 53 and the Bern Illegitimate Task Scale. 54 We included an item based on the latest definition of “Excessive DocBurden” to explore the relevance of the task to patient care. 25 We adapted the items from original instruments to improve survey comprehension, resulting in a total of 12 statements: Four related to usability, five to task burden, two to task legitimacy, and one to task relevance to care delivery. The instrument comprised five sections: (1) Time spent/activity, (2) Task burden ranking, (3) Evaluation of six individual documentation tasks 27 38 39 using 11 items on a six-point Likert scale (i.e., Very Strongly Disagree to Very Strongly Agree), (4) Open-ended questions, and (5) Demographic questions. Content and face validity were established a priori through item derivation from three well-validated parent instruments, along with structured feedback from 10 providers (physicians and APPs) during pilot testing, resulting in the refinement of the instrument to 11 items before full-scale deployment. Cronbach's α was 0.86 for the 11-item scale, ranging from 0.79 (poor usability) to 0.83 (task value) across the three factors. Composite reliability (Raykov's *r* ) values were 0.80 to 0.85, exceeding the 0.70 criterion for acceptable internal consistency. 55 Survey length was 111 questions; median duration was 11.5 minutes. Of the 104 qualified respondents who opened the survey, 28% exited before completion, with a median elapsed time before attrition of 13.8 minutes.

### Data Analysis

Statistical analysis was performed using SPSS version 29. Of the 153 persons who accessed the link, 61 respondents (40%) provided complete data, which were screened; variations were addressed, and responses to the two negative questions were reverse-coded. Associations between provider demographics and documentation burden outcomes were examined in three steps. First, a multivariate analysis of variance (MANOVA) was run with each item entered simultaneously as a fixed factor and the seven burden scores as the dependent variable vector (Pillai's Trace criterion). When a multivariate term reached significance (α = 0.05), we decomposed the effect with univariate one-way ANOVAs for each burden domain, applying Bonferroni adjustment to control family-wise error. For qualitative analyses, narratives were evaluated for general sentiment and coded as agreeing, neutral, or disagreeing. 56 No discrepancies were noted in interrater agreement on the coding of positive responses. Logistic regression and chi-square tests were applied to categorical variables.

EFA identified underlying relationships among multiple items, aligning with the intent to develop a conceptual framework of clinical documentation burden. Given the limited sample size, researchers extracted the document task for each participant with the highest-burden rank scores (Kaiser–Meyer–Olkin [KMO] 0.704; Bartlett's *p* < 0.001) to improve the factor-to-participant ratio. The EFA was conducted using principal axis factoring as the extraction method. To find the adequate number of factors, the Kaiser–Guttman criterion approach was used, 57 then compared with the scree-plot method. 58 Oblimin rotation was chosen to obtain a transformed factor loading matrix that allowed a more straightforward assignment of items to correlated factors (see Supplementary Appendix C [available in the online version only]). 59

## Results

Survey respondents were predominantly female (70.5%), with the majority being 35 to 54 years old (54.1%), followed by 23% aged 25 to 34, and having more than 20 years of experience (34.4%). The most represented were APRNs (55.7%), followed by physicians (27.9%), then PAs (13.1%). Most participants work in either a hospital setting (49.2%) or in ambulatory care (44.3%), with approximately 74% reporting an academic affiliation ( Table 1 ).

**Table 1:** Characteristics of clinical documentation burden study participants

Characteristics	*n* = 61
Age (y)	
25–34	14 (23.0)
35–44	17 (27.9)
45–54	16 (26.2)
55–64	8 (13.1)
≥65	5 (8.2)
Unknown	1 (1.6)
Gender	
Female	43 (70.5)
Male	17 (27.9)
Unknown	1 (1.6)
Role	
APRN	34 (55.7)
Physician	17 (27.9)
Physician assistant	8 (13.1)
Resident	1 (1.6)
Other	1 (1.6)
Specialty	
Medical specialty	25 (41.0)
Other specialty	15 (24.6)
Primary care/generalist	12 (19.7)
Surgical specialty	8 (13.1)
Unknown	1 (1.6)
Experience (years)	
<5	10 (16.4)
6–10	11 (18.0)
11–15	9 (14.8)
16–20	10 (16.4)
>20	21 (34.4)
Education	
Master's degree	36 (59.0)
Doctorate or professional degree	24 (39.3)
Unknown	1 (1.6)
Clinical hours, per week	
21–30	3 (4.9)
31–40	16 (26.2)
41–50	21 (34.4)
51–60	16 (26.2)
61–70	3 (4.9)
71–80	1 (1.6)
>81	1 (1.6)
Setting	
Hospitals	30 (49.2)
Ambulatory care settings	27 (44.3)
Long-term care facilities	1 (1.6)
Private or group practice	1 (1.6)
Rehabilitation centers	1 (1.6)
Urgent care centers	1 (1.6)
Academic affiliation	
Yes	45 (73.8)

Abbreviation: APRN, advance practice registered nurse.

Note: All values expressed as *n* (percentage).

### Descriptive Statistics

**Fig. 1:**
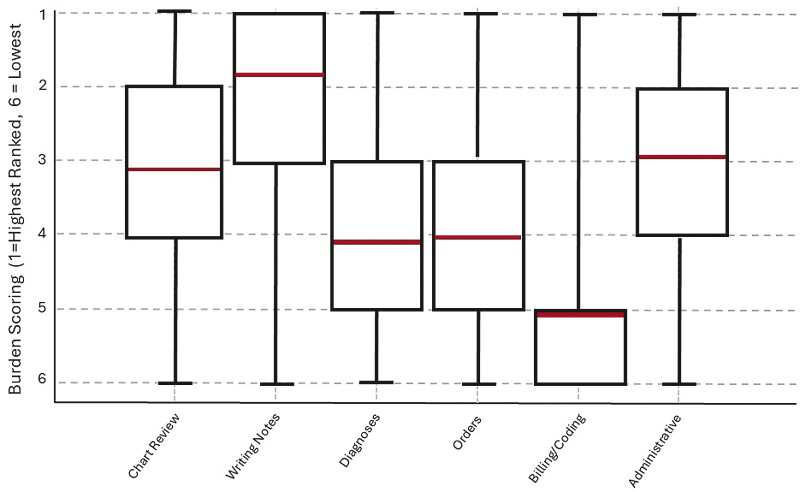
Distribution of six key documentation tasks after being ranked from most to least burdensome.

Given the overrepresentation of APPs, role-specific results are summarized descriptively ( Table 2 ) to provide transparency on potential group-level differences. When respondents globally ranked all documentation activity categories from most to least burdensome (1 = first ranked and 6 = lowest ranked), the most burdensome mean score was for writing notes (1.84 ± 1.48). In contrast, billing and coding tasks were considered the least burdensome (5.03 ± 1.46; Table 3 and Fig. 1 ). The category of administrative tasks demonstrated the highest variability in responses, while writing notes showed the least. The average time spent on tasks was highest for writing notes (3.12 hours/shift, SD 1.59) and lowest for billing/coding (0.63 hours/shift, SD 0.48 hours). Most burdensome tasks showed stronger agreement (higher means, negative skew), suggesting that providers have stronger, more unified opinions about what makes a documentation task more, rather than less, burdensome.

**Table 2:** Comparison of burden globally ranked for six key documentation tasks categorized by provider role

	All roles	Physicians	APRNs	PAs	Other roles
	*n* = 61	*n* = 17	*n* = 34	*n* = 8	*n* = 2
Review	3.10 (1.46)	3.65 (1.37)	2.97 (1.53)	2.25 (0.89)	4.00 (1.41)
Notes	1.84 (1.23)	2.06 (1.43)	1.82 (1.24)	1.50 (0.76)	1.50 (0.71)
Problem	4.08 (1.31)	4.47 (1.70)	3.94 (1.23)	3.88 (0.64)	4.00 (0)
Orders	4.02 (1.28)	3.65 (1.50)	4.15 (1.26)	4.25 (0.89)	4.00 (1.41)
Billing	5.03 (1.48)	4.59 (1.28)	4.97 (1.68)	6.00 (0)	6.00 (0)
Admin	2.93 (1.52)	2.59 (1.58)	3.15 (1.44)	3.13 (1.73)	1.50 (0.71)

Abbreviations: APRN, advance practice registered nurse; PA, physician assistant.

Note: All values expressed as mean (standard deviation); 1 = highest ranked burden versus 6 = lowest ranked burden.

**Table 3:** Descriptive statistics of ranked burden across six key documentation tasks for all health care provider roles

	Review	Notes	Problems	Orders	Billing	Admin
Mean	3.10	1.84	4.08	4.02	5.03	2.93
Standard error	0.19	0.16	0.17	0.16	0.19	0.19
Median	3.00	1.00	4.00	4.00	6.00	3.00
Mode	2.00	1.00	4.00	5.00	6.00	3.00
Standard deviation	1.46	1.23	1.31	1.28	1.48	1.52
Sample variance	2.12	1.51	1.71	1.65	2.20	2.30
Minimum	1.00	1.00	1.00	2.00	1.00	1.00
Maximum	6.00	6.00	6.00	6.00	6.00	6.00

Note: 1 = highest ranked burden versus 6 = lowest ranked burden.

Although responses were obtained for all six specific tasks individually, this paper examines the data from the documentation tasks rated as most burdensome by each respondent ( Table 4 ). Correlation analysis of individual Likert items revealed several significant relationships. The strongest inter-item correlations were related to documentation value, with the strongest relationship between unnecessary documentation and perceived relevance ( *r* = 0.854), followed by being considered unreasonable ( *r* = 0.783). Unreasonable documentation requirements were similarly associated with lack of relevance ( *r* = 0.727), suggesting a clear cluster of items related to documentation value perception.

**Table 4:** Descriptive statistics of 11 items for most burdensome documentation tasks for all health care providers

	Mean	SD	Min	Max
Q1. Performing this task is unnecessarily complex	4.33	1.38	1.00	6.00
Q2. Performing this task is [not] easy to do	4.30	1.13	2.00	6.00
Q3. The necessary functions to complete this task are [not] well-integrated	4.28	1.19	2.00	6.00
Q4. There is too much inconsistency when trying to complete this task	3.82	1.38	1.00	6.00
Q5. An excessive amount of mental activity is required to perform this task	4.44	1.13	1.00	6.00
Q6. An excessive amount of physical activity is required to complete this task	4.30	1.19	1.00	6.00
Q7. I have to work very hard (mentally and physically) to perform this task	4.23	1.32	1.00	6.00
Q8. I feel very irritated, stressed, or annoyed during this task	4.48	1.16	1.00	6.00
Q9. This task feels unnecessary	3.03	1.45	1.00	6.00
Q10. This task feels unreasonable	3.18	1.36	1.00	6.00
Q11. This task is not relevant to improving patient care or outcomes	2.90	1.35	1.00	6.00

Abbreviation: SD, standard deviation.

A second cluster emerged around system integration and mental demands. The item related to poor system integration showed a strong correlation with mental load ( *r* = 0.719), and mental load was strongly correlated with the need to work hard ( *r* = 0.690), suggesting that systems perceived as poorly integrated may contribute to an increased cognitive burden. A third cluster involved system complexity and ease of use. System complexity showed moderate correlations with poor integration ( *r* = 0.557) and lack of ease ( *r* = 0.537), suggesting that more complex systems are perceived as less integrated and more challenging to use.

These correlations suggest that documentation burden manifests in interconnected ways, with system complexity, perceived integration issues, and perceived value of documentation requirements forming distinct but related aspects of the overall burden experience. Analysis of positively coded sentiments ( Table 5 ) revealed that practitioners experiencing PD were more likely to rate documentation tasks as unnecessarily complex, less easy, and poorly integrated. Additionally, these individuals reported higher mental activity and greater irritation associated with documentation activities. Increased physical load and work intensity were linked with a greater likelihood of burnout ( Table 6 ).

**Table 5:** Summary of narrative responses to open questions about the impact of clinical documentation burden

Characteristics	*n* = 61
Does the burden of clinical documentation create a conflict between your personal values as a caregiver and the values of the health care system?	
Affirmative	44 (72.1)
Negative	10 (16.4)
Neutral	6 (10.0)
Do clinical documentation demands impact your emotional well-being at work?	
Affirmative	46 (75.4)
Negative	10 (16.4)
Neutral	4 (6.6)

Note: All values expressed as *n* (percentage).

**Table 6:** Correlations between professional dissonance and burnout with individual survey items

Items	Professional dissonance ( *n* = 44)	Burnout ( *n* = 46)
Q1. Unnecessarily complex	**0.284 (** ***p*** ** = 0.027)**	0.103 ( *p* = 0.431)
Q2. Not easy to do	**0.327 (** ***p*** ** = 0.010)**	0.144 ( *p* = 0.269)
Q3. Functionality not well-integrated	**0.427 (** ***p*** ** < 0.001)**	0.129 ( *p* = 0.320)
Q4. Too much inconsistency	−0.055 ( *p* = 0.674)	0.013 ( *p* = 0.918)
Q5. Excessive mental activity	**0.310 (** ***p*** ** = 0.015)**	0.215 ( *p* = 0.096)
Q6. Excessive physical activity	0.218 ( *p* = 0.092)	**0.236 (** ***p*** ** = 0.047)**
Q7. Work very hard	0.286 ( *p* = 0.052)	**0.352 (** ***p*** ** = 0.007)**
Q8. Feel irritated or stressed	**0.415 (** ***p*** ** < 0.001)**	0.225 ( *p* = 0.081)
Q9. Feels unnecessary	0.167 ( *p* = 0.199)	0.094 ( *p* = 0.472)
Q10. Feels unreasonable	0.219 ( *p* = 0.091)	0.073 ( *p* = 0.576)
Q11. Not relevant to care/outcomes	0.145 ( *p* = 0.263)	0.018 ( *p* = 0.890)

Note: All values expressed as *r* ( *p* -value). Bold values are statistically significant.

Exploring the influence of individual characteristics on perceptions of documentation burden revealed several significant correlations. The omnibus MANOVA was significant (Pillai's Trace = 0.71, *F* [21, 150] = 2.26, *p* = 0.004), indicating that at least one provider characteristic was associated with the combined burden measures. Subsequent univariate ANOVAs revealed significant demographic variations in documentation burden. Age displayed a weak-to-moderate positive relationship with perceiving documentation as unnecessary ( *r* = 0.239, *p* = 0.023) and irrelevant ( *r* = 0.286, *p* = 0.006); older respondents, therefore, tended to rate paperwork as less essential. Male sex also correlated moderately with an elevated perceived sense of burden (unnecessary: *r* = 0.342, *p* < 0.001; irrelevant: *r* = 0.346, *p* < 0.001). Primary-care/generalist affiliation was moderately associated with rating documentation as difficult (not easy: *r* = 0.389, *p* < 0.001; complex: *r* = 0.238, *p* = 0.024), whereas surgical affiliation showed a weak inverse relationship with irritation ( *r* = −0.286, *p* = 0.007). Primary-care clinicians had approximately three times the odds of reporting PD compared with subspecialists (OR = 3.12, 95% CI 1.17–8.29, *p* = 0.024).

Age-specific burnout patterns emerged, with the highest rates in the 25 to 34 group (85.7%, lowest PD at 57.1%) compared with the 65+ group (60% burnout, 80% PD). Experience influenced perceptions: years in practice were weakly, inversely related to viewing documentation as unreasonable (16–20 years, *r* = −0.217, *p* = 0.040) and to irritation among those practicing less than 5 years ( *r* = −0.214, *p* = 0.043). Educational attainment was correlated with perceived system integration issues: Possessing a doctorate was positively associated ( *r* = 0.316, *p* = 0.018), whereas having a master's degree was negatively associated ( *r* = −0.316, *p* = 0.018).

### Exploratory Factor Analysis

The EFA revealed three factors with eigenvalues greater than 1.0, which were confirmed by observation of the scree plot. These three factors explained approximately 70% of the total variance of the 11 items when looking at the highest-ranked tasks. 60 Each factor was required to have at least three moderate-to-strong loadings—factor loadings of at least 0.40 were considered to be satisfactory. 61 62 For tasks with the highest burden ranking, there were three distinct constructs, with items loading on Factor 1 (F1) all related to poor usability, those on F2 related to perceived high task value, and items loading on F3 related to excessive mental exertion ( Table 7 ). These factors were screened for conceptual clarity and redundancy to retain only the most distinct and informative items. All items showed a clear theoretical link with the target construct of clinical documentation burden.

**Table 7:** Exploratory factor analysis with item attribution for most burdensome documentation tasks

Name of factor		Item content	F1	F2	F3
Poor usability (Factor 1)	Q1	Performing this task is unnecessarily complex	**0.657**	−0.044	0.032
	Q2R	Performing this task is [not] easy to do	**0.640**	−0.003	0.101
	Q3R	The necessary functions to complete this task are [not] well-integrated	**0.899**	0.076	−0.148
	Q6	An excessive amount of physical activity (clicking, scrolling, transitioning between screens, etc.) is required to complete this task	**0.438**	0.121	0.285
Perceived high task value (Factor 2)	Q9	This task feels unnecessary	−0.001	**−0.987**	−0.109
	Q10	This task feels unreasonable	0.155	**−0.791**	0.020
	Q11	This task is not relevant to improving patient care or outcomes	−0.177	**−0.938**	0.032
Excessive mental exertion (Factor 3)	Q5	An excessive amount of mental activity (concentration, recall, etc.) is required to perform this task	−0.037	0.080	**0.892**
	Q7	I have to work very hard (mentally and physically) to perform this task	−0.027	−0.015	**0.881**
	Q8	I feel very irritated, stressed, or annoyed during this task	0.281	−0.330	**0.435**
		Sums of squared loadings	3.418	2.354	1.025
		Percentage of variance	31.07	21.40	9.32
		Cumulative percentage	31.07	52.47	61.79

Note: Bold values are statistically significant.

Pearson correlations revealed that F1 (poor usability; 4 items) showed a weak but significant negative correlation with F2 (perceived high task value; 3 items; *r* = −0.270; *p* = 0.042), and a moderate correlation with F3 (excessive mental exertion; 3 items; *r* = 0.450; *p* < 0.001). F2 and F3 were negatively correlated and were not significant ( *r* = −0.008, *p* = 0.952). No correlations exceeded *r* = 0.70, suggesting no problems with multicollinearity. 63 Analyses revealed several significant relationships between factors and the coded qualitative sentiments. A positive response to the PD question was significantly correlated with F1 ( *r* = 0.377, *p* = 0.004) and F3 ( *r* = 0.362, *p* = 0.006) but not with F2 ( *r* = −0.205, *p* = 0.125). A positive response to the burnout question was significantly correlated with F3 ( *r* = 0.314, *p* = 0.017) but not F1 ( *r* = 0.191, *p* = 0.154) or F2 ( *r* = −0.129, *p* = 0.339). A positive response to the PD question showed a significant correlation with agreement with the burnout question ( *r* = 0.384, *p* = 0.002). Both parametric and non-parametric correlation approaches yielded similar interpretations, indicating that the associations are robust and independent of the method.

### Conceptual Framework of Clinical Documentation Burden

**Fig. 2:**
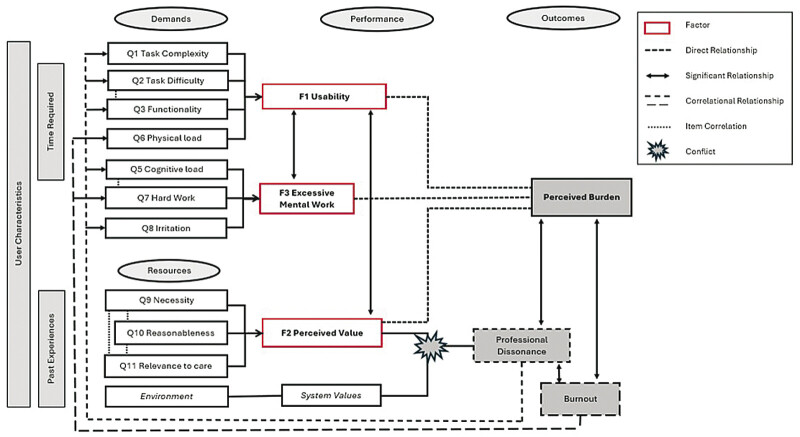
Preliminary conceptual framework of clinical documentation burden.

The theoretical conceptual diagram seeks to illustrate the relationships between components discovered during analysis ( Fig. 2 ). Initial elements include the individual items queried in the instrument (Q1–Q11), which are then attributed to latent factors (F1–F3) that reflect underlying patterns in the theoretical concept of perceived documentation burden. F2 contributes to a clearer understanding of the role of PD and burnout in the context of documentation burden. Supported by significant correlations, professional burden, and burnout outcomes are then linked back to the individual items they influence, which affect the contribution of the three factors.

At its core, the framework posits that heightened task demands are influenced by the usability of documentation systems and the extent of mental effort required, which can precipitate adverse outcomes such as perceived burden, PD, and ultimately, burnout. Usability serves as a mediator in this pathway; when systems are user-friendly and efficiently designed, the detrimental effects of high-task demands can be mitigated. Furthermore, the model recognizes the pivotal role of both personal and environmental resources as moderating agents. Task value, informed by the perceived necessity and relevance of the tasks, helps buffer the strain induced by demanding tasks. Simultaneously, the work environment, as embodied in factors such as organizational values, further modulates the relationship between perceived task value and burden. Internal personal resources facilitate engagement and are believed to cushion the adverse effects when demands are intense.

The model builds upon the fundamental principles of the Job Demands-Resources (JD-R) theory. The theory's central tenet states that balancing demands with adequate resources is essential for maintaining well-being and achieving optimal performance. 64 Mapping clinical documentation demands to job demands and introducing perceived value as a crucial resource highlights how demands as potential stressors can lead to adverse outcomes if not balanced by adequate resources to buffer their effect. Like the JD-R framework, which values personal resources and organizational resources, the model incorporates individual factors (e.g., perceived necessity and relevance) and system-level values. In the context of clinical documentation, the model's outcomes mirror the negative consequences outlined in the JD-R framework when demands exceed available resources.

Another theoretical framework that influences the model is the Expectancy Value Model of Motivation, which contributes a focus on personal incentive, specifically the value placed on a task. It argues that individuals are more motivated to perform a task and assumedly less burdened in doing so if they value the success, considering the cost or demand. 33 Cognitive Load Theory (CLT) has been reflected in a shared concern with managing cognitive demands to optimize performance and well-being. 22 Our model, like CLT, distinguishes between intrinsic load (task complexity), extraneous load (inefficient task design), and germane load (resources devoted to meaningful engagement) by emphasizing usability as a mediator that reduces extraneous demands and optimizes task performance. The final theory referenced in this model is PD, which accurately describes the dynamic when the perceived value factor and the system values are not in alignment. 47

Our model allows for feedback loops where the experienced outcomes may re-evaluate and influence future perceptions of task demands and the adequacy of both personal and environmental resources. This dynamic system emphasizes that while effective resource allocation can attenuate the effect of high demands, a chronic imbalance may lead to progressively adverse outcomes. The model clarifies the causal pathways that underpin documentation burden and highlights potential levers for intervention. By optimizing usability and strategically clarifying the importance and relevance of documentation tasks, it is possible to counteract the consequences of excessive task demands and promote better overall outcomes in clinical documentation settings.

## Discussion

This study revealed three meaningful factors—poor usability, perceived task value, and excessive mental exertion. This structure highlights the intricate interplay between design challenges, task engagement, and cognitive load, indicating that usability issues compromise user experience and are intertwined with increased mental effort, ultimately contributing to a shift in perceived task value. These findings should be interpreted in light of the APP-heavy sample, underscoring the exploratory and preliminary nature of the framework.

Poor usability was found to have a strong correlation with excessive mental exertion, aligning with previous research. 65 66 67 The negative correlation between poor usability and perceived task value reflects effort justification to resolve cognitive dissonance and PD—as users encounter more usability issues, they tend to value the task less. 68 Usability issues were significantly correlated with PD, indicating that interface problems may contribute to users feeling disconnected from their work. However, poor usability did not correlate with burnout, suggesting that usability issues alone may not lead to burnout without the mediating effect of mental exertion. Excessive mental exertion showed significant correlations with PD and burnout, suggesting psychological costs of sustained cognitive demands, 10 and corroborating findings by Shanafelt et al. 2

A noteworthy finding is the significant relationship between PD and documentation burden. Providers who frequently experience PD more often reported their documentation tasks as unnecessarily complex and poorly integrated, suggesting that the misalignment between professional expectations and daily documentation requirements may be more profound than previously reported. The strong correlation between perceived work effort and burnout aligns with previous research but adds a crucial dimension specific to the documentation burden. The findings regarding mental load and documentation task-related irritation levels suggest that the effect extends beyond inconvenience to potentially affect provider cognitive capacity and emotional well-being. This has implications for patient safety and quality of care, as cognitive overload has been linked to an increased incidence of medical errors. 10

Each factor suggests a different intervention lever: (1) Poor usability—interface re-design and automation; (2) Low task value—policy revision to remove low-value documentation; (3) Excessive mental exertion—workflow re-sequencing or ambient intelligence tools to offload cognitive steps. Linking burden profiles to specific levers can help organizations prioritize high-yield fixes over generic training alone. Thus, the study's chief contribution is not a finished tool but a conceptual and methodological blueprint that moves the field toward a repeatable, psychometrically sound approach for quantifying documentation burden and evaluating targeted solutions. Physician-specific tasks such as teaching, supervision, and research were not captured and may introduce additional burden dimensions not represented here. The study's novelty lies not in rediscovering burden factors but in empirically testing their integration into a theory-driven framework, positioning this as a foundation for future cumulative science.

### Limitations

Several limitations of this study warrant consideration, the first of which is the sample size. Although the overall sample size ( *N* = 61) limits sensitivity for omnibus comparisons, post hoc power analysis shows that the study retained more than 99% power to detect the moderate-to-large correlations observed (e.g., burnout vs. documentation time, *r* = 0.35). Conversely, for medium effects in a four-comparison group (provider types) MANOVA framework, the achieved power was 0.32, indicating an increased risk of Type II error for small-to-medium group differences. The primary relationships of interest—moderate correlations among burden and other variables—are robustly powered. The sample yields a 5.5:1 participant-to-item ratio; simulation work shows stable three-factor recovery at a ratio of at least 5:1 with communalities greater than 0.6. The KMO of 0.704 in this study, therefore, supports our preliminary interpretation. 69 However, validation of this factor structure in a larger, independent sample via confirmatory factor analysis (CFA) is warranted.

Second, the overrepresentation of female providers (71%), 70 APPs (56%), and those with academic affiliations (74%) may limit the generalizability of the findings to other practice settings and provider demographics. 71 This sample was dominated by APPs, limiting physician-specific insights; subgroup descriptive data are presented ( Table 2 ), but adequately powered role-based comparisons will require larger samples. Therefore, future studies should deliberately recruit larger and more balanced samples to allow role-based comparisons and subgroup validation.

Third, survey length (111 items) and voluntary participation may have introduced selection bias, as individuals with greater tolerance for computer-based tasks or more available time may have been more likely to complete the instrument. Although the median completion time was 11.5 minutes and internal reliability was strong (Cronbach's α = 0.86), future refinements should streamline the survey to reduce the burden on respondents.

Fourth, important contextual factors such as the use of scribes or efficiency tools like ambient generative artificial intelligence (AI), patient panel size, and case complexity were not assessed. These variables are likely to influence the perception of documentation burden and should be incorporated into future iterations of the survey. Finally, the cross-sectional nature of the study prevents causal inference between documentation burden and provider burnout. Finally, the model's reliance on self-reported data may have introduced bias and would need to be validated with a more robust, CFA.

## Conclusion

Collectively, these findings underscore the critical effect of documentation burden on health care providers. They suggest that interventions aimed at refining documentation processes alleviate the operational strain and potentially mitigate PD and burnout, thereby enhancing overall patient care. The theoretical contribution of this study lies in the development of a preliminary model that integrates psychological factors, technology, and clinical documentation attributes to elucidate the concept of clinical documentation burden. The contribution of this study is not simply in re-identifying common burden factors; its novelty lies in developing a preliminary, theory-driven conceptual framework that explicitly maps usability, perceived task value, and mental exertion to well-established related psychological models.

This study deliberately combines items adapted from three widely validated instruments—SUS, NASA-TLX, and Bern Illegitimate Task Scale—into a single, theory-informed survey. Although that adaptation is not itself a fully standardized tool, the resulting trifactor exploratory conceptual framework offers a scaffold for future synchronization of efforts. Specifically, the emergent dimensions of poor usability, perceived task value, and excessive mental exertion map onto the Job Demands-Resources model (demands vs. resources), Expectancy-Value theory (utility and cost), and CLT (intrinsic vs. extraneous load). By making these latent constructs explicit and psychometrically distinct, the model clarifies what should be measured across studies—not merely how long documentation takes.

### Future Research

Building on this preliminary evidence, future work will include a CFA and test–retest study seeking to develop a Clinical Documentation Burden Inventory that retains the three-factor cores, adding additional anchor items to improve reliability. If the factor structure is replicated, this instrument would serve as a common outcomes measure analogous to the Maslach Burnout Inventory, enabling meta-analytic synthesis and direct benchmarking of interventions. Future research should focus on empirical studies to validate this framework, ensuring its applicability across diverse health care settings and its effectiveness in reducing documentation burden.

## Clinical Relevance Statement

The findings highlight the significant effect of documentation burden on health care providers, suggesting that refining documentation processes to better align with individual psychological attributes could alleviate operational strain and reduce PD and burnout. This could enhance overall patient care outcomes by improving provider well-being and efficiency. Practitioners and health care organizations should consider interventions that incorporate perceived task value to enhance provider satisfaction and patient safety.

## Multiple-Choice Questions

1. Which factor was identified as having a strong positive correlation with excessive mental exertion in the study of clinical documentation burden?
  - Perceived task value
  - Poor usability
  - Workflow integration
  - Task relevance
  **Correct Answer** : The correct answer is option b. The study found a strong positive correlation between poor usability and excessive mental exertion. This suggests that when documentation systems are not user-friendly, they require more mental effort from health care providers, contributing to the overall burden. Usability issues can lead to increased cognitive load, making tasks more demanding and potentially leading to burnout if not addressed.
2. What is a key component of the conceptual model developed in the study to address clinical documentation burden?
  - Task automation
  - Professional dissonance
  - Patient feedback
  - Financial incentives
  **Correct Answer** : The correct answer is option b. The preliminary conceptual framework developed in the study incorporates PD as a key component. This concept refers to the conflict that arises when health care providers' values do not align with the demands of their documentation tasks. The model suggests that addressing this dissonance by aligning tasks with providers' values can help reduce the perceived burden and improve job satisfaction and patient care outcomes.
3. According to the study, what role does usability play in the experience of documentation burden?
  - It acts as a barrier to task completion.
  - It serves as a mediator in the pathway to burnout. (Correct)
  - It is irrelevant to task performance.
  - It solely affects physical exertion.
  **Correct Answer** : The correct answer is option b. Usability is identified as a mediator in the pathway to burnout within the conceptual framework. When documentation systems are user-friendly and efficiently designed, they can mitigate the adverse effects of high task demands. This reduces the cognitive load on health care providers, thereby decreasing the likelihood of burnout and improving overall task performance and satisfaction.
