# Supplementary material for: A Preliminary Conceptual Framework of Clinical Documentation Burden: Exploratory Factor Analysis Investigating Usability, Effort, and Perceived Burden among Health Care Providers
Source: Appl Clin Inform. 2025 Dec 12;16(5):1815–27. doi: 10.1055/a-2751-1896 (PMC12700715; doi:10.1055/a-2751-1896)
Supplement: Supplementary file 1 — Supplementary Material [file 10-1055-a-2751-1896_27710725.pdf]

Understanding Clinical Documentation Burden

Study Title: Exploring Clinical Documentation Burden: A Study on Usability, Effort, and Perceived Burden Among

Healthcare Providers

This 10-minute survey aims to explore the usability, effort, and perceived burden of clinical documentation among healthcare providers. By gathering these insights, researchers will be better able to support future improvements to reduce clinician burnout

MUSC IRB-Pro00140782: View IRB Approved Information Sheet

Having read the information above, do you voluntarily agree to participate in this research study?	◯ Yes<br>◯ No
Are you a healthcare provider currently involved in providing clinical care in the United States?	◯ Yes<br>◯ No

We appreciate your interest in participating! Unfortunately, at this time, we are focused on learning more about providers who are currently engaged in delivering clinical care.

Applied Clinical Informatics | DOI 10.1055/a-2751-1896 | © The Author(s).

Understanding Clinical Documentation Burden

Thank you for agreeing to support our efforts to better understand clinical documentation burden.

The purpose of this survey is to gather insights into the perceived burden associated with various documentation activities in your daily workflow as a provider. Your responses will help us to evaluate the complexity, consistency, and relevance of these tasks to patient care and your professional role.

projectredcap.org REDCap

Time Spent This section asks you to estimate how long you spent each day/shift on different types of documentation activities.

How much time do you typically spend per day/shift on: Accessing the system (i.e. signing into EHR)?

____
(Please answer in half hour (0.5) increments)

How much time do you typically spend per day/shift on: Chart Review?

____
(Please answer in half hour (0.5) increments)

How much time do you typically spend per day/shift on: Writing Notes?

____
(Please answer in half hour (0.5) increments)

How much time do you typically spend per day/shift on: Addressing Problem/Diagnosis Lists?

____
(Please answer in half hour (0.5) increments)

How much time do you typically spend per day/shift on: Order Entry?

____
(Please answer in half hour (0.5) increments)

How much time do you typically spend per day/shift on: Billing/Coding?

____
(Please answer in half hour (0.5) increments)

How much time do you typically spend per day/shift on: Administrative Tasks (i.e. Prior Authorization, In Basket communications)?

____
(Please answer in half hour (0.5) increments)

Burden Ranking Please rank the following activities from most to least burdensome, with 1 being the most amount of burden and 8 being the least amount of burden

	1 Most	2	3	4	5	6	7 Least
Accessing the system	○	○	○	○	○	○	○
Chart Review	○	○	○	○	○	○	○
Writing Notes	○	○	○	○	○	○	○
Addressing Problem/Diagnosis Lists	○	○	○	○	○	○	○
Order Entry	○	○	○	○	○	○	○
Billing/Coding	○	○	○	○	○	○	○
Administrative Tasks	○	○	○	○	○	○	○

Applied Clinical Informatics | DOI 10.1055/a-2751-1896 | © The Author(s).

Task Evaluation Please read each statement below carefully. These statements relate to your experience with a series of documentation tasks. By asking about each task separately, we aim to better understand how specific tasks are more burdensome than others.

For each statement, indicate your level of agreement by selecting the option that best reflects your opinion

Task: Writing Notes

	Very Strongly Disagree	Strongly Disagree	Disagree	Agree	Strongly Agree	Very Strongly Agree
Performing this task is unnecessarily complex	○	○	○	○	○	○
Performing this task is easy to do	○	○	○	○	○	○
The necessary functions to complete this task are well-integrated	○	○	○	○	○	○
There is too much inconsistency when trying to complete this task	○	○	○	○	○	○
An excessive amount of mental activity (concentration, recall, etc.) is required to perform this task	○	○	○	○	○	○
An excessive amount of physical activity (clicking, scrolling, transitioning between screens, etc.) is required to complete this task	○	○	○	○	○	○
I have to work very hard (mentally and physically) to perform this task	○	○	○	○	○	○
I feel very irritated, stressed, or annoyed during this task	○	○	○	○	○	○
This task feels unnecessary	○	○	○	○	○	○
This task feels unreasonable	○	○	○	○	○	○
This task is not relevant to improving patient care or outcomes	○	○	○	○	○	○

Applied Clinical Informatics | DOI 10.1055/a-2751-1896 | © The Author(s).

Task Evaluation For each statement, indicate your level of agreement by selecting the option that best reflects your opinion

Task: Chart Review

	Very Strongly Disagree	Strongly Disagree	Disagree	Agree	Strongly Agree	Very Strongly Agree
Performing this task is unnecessarily complex	○	○	○	○	○	○
Performing this task is easy to do	○	○	○	○	○	○
The necessary functions to complete this task are well-integrated	○	○	○	○	○	○
There is too much inconsistency when trying to complete this task	○	○	○	○	○	○
An excessive amount of mental activity (concentration, recall, etc.) is required to perform this task	○	○	○	○	○	○
An excessive amount of physical activity (clicking, scrolling, transitioning between screens, etc.) is required to complete this task	○	○	○	○	○	○
I have to work very hard (mentally and physically) to perform this task	○	○	○	○	○	○
I feel very irritated, stressed, or annoyed during this task	○	○	○	○	○	○
This task feels unnecessary	○	○	○	○	○	○
This task feels unreasonable	○	○	○	○	○	○
This task is not relevant to improving patient care or outcomes	○	○	○	○	○	○

Applied Clinical Informatics | DOI 10.1055/a-2751-1896 | © The Author(s).

Task Evaluation For each statement, indicate your level of agreement by selecting the option that best reflects your opinion

Task: Administrative Tasks

	Very Strongly Disagree	Strongly Disagree	Disagree	Agree	Strongly Agree	Very Strongly Agree
Performing this task is unnecessarily complex	○	○	○	○	○	○
Performing this task is easy to do	○	○	○	○	○	○
The necessary functions to complete this task are well-integrated	○	○	○	○	○	○
There is too much inconsistency when trying to complete this task	○	○	○	○	○	○
An excessive amount of mental activity (concentration, recall, etc.) is required to perform this task	○	○	○	○	○	○
An excessive amount of physical activity (clicking, scrolling, transitioning between screens, etc.) is required to complete this task	○	○	○	○	○	○
I have to work very hard (mentally and physically) to perform this task	○	○	○	○	○	○
I feel very irritated, stressed, or annoyed during this task	○	○	○	○	○	○
This task feels unnecessary	○	○	○	○	○	○
This task feels unreasonable	○	○	○	○	○	○
This task is not relevant to improving patient care or outcomes	○	○	○	○	○	○

Task Evaluation For each statement, indicate your level of agreement by selecting the option that best reflects your opinion

Task:Addressing Problem/Diagnosis Lists

	Very Strongly Disagree	Strongly Disagree	Disagree	Agree	Strongly Agree	Very Strongly Agree
Performing this task is unnecessarily complex	◯	◯	◯	◯	◯	◯
Performing this task is easy to do	◯	◯	◯	◯	◯	◯
The necessary functions to complete this task are well-integrated	◯	◯	◯	◯	◯	◯
There is too much inconsistency when trying to complete this task	◯	◯	◯	◯	◯	◯
An excessive amount of mental activity (concentration, recall, etc.) is required to perform this task	◯	◯	◯	◯	◯	◯
An excessive amount of physical activity (clicking, scrolling, transitioning between screens, etc.) is required to complete this task	◯	◯	◯	◯	◯	◯
I have to work very hard (mentally and physically) to perform this task	◯	◯	◯	◯	◯	◯
I feel very irritated, stressed, or annoyed during this task	◯	◯	◯	◯	◯	◯
This task feels unnecessary	◯	◯	◯	◯	◯	◯
This task feels unreasonable	◯	◯	◯	◯	◯	◯
This task is not relevant to improving patient care or outcomes	◯	◯	◯	◯	◯	◯

Applied Clinical Informatics | DOI 10.1055/a-2751-1896 | © The Author(s).

Task Evaluation For each statement, indicate your level of agreement by selecting the option that best reflects your opinion

Task: Order Entry

	Very Strongly Disagree	Strongly Disagree	Disagree	Agree	Strongly Agree	Very Strongly Agree
Performing this task is unnecessarily complex	○	○	○	○	○	○
Performing this task is easy to do	○	○	○	○	○	○
The necessary functions to complete this task are well-integrated	○	○	○	○	○	○
There is too much inconsistency when trying to complete this task	○	○	○	○	○	○
An excessive amount of mental activity (concentration, recall, etc.) is required to perform this task	○	○	○	○	○	○
An excessive amount of physical activity (clicking, scrolling, transitioning between screens, etc.) is required to complete this task	○	○	○	○	○	○
I have to work very hard (mentally and physically) to perform this task	○	○	○	○	○	○
I feel very irritated, stressed, or annoyed during this task	○	○	○	○	○	○
This task feels unnecessary	○	○	○	○	○	○
This task feels unreasonable	○	○	○	○	○	○
This task is not relevant to improving patient care or outcomes	○	○	○	○	○	○

Applied Clinical Informatics | DOI 10.1055/a-2751-1896 | © The Author(s).

Task Evaluation For each statement, indicate your level of agreement by selecting the option that best reflects your opinion

Task: Accessing the System

	Very Strongly Disagree	Strongly Disagree	Disagree	Agree	Strongly Agree	Very Strongly Agree
Performing this task is unnecessarily complex	○	○	○	○	○	○
Performing this task is easy to do	○	○	○	○	○	○
The necessary functions to complete this task are well-integrated	○	○	○	○	○	○
There is too much inconsistency when trying to complete this task	○	○	○	○	○	○
An excessive amount of mental activity (concentration, recall, etc.) is required to perform this task	○	○	○	○	○	○
An excessive amount of physical activity (clicking, scrolling, transitioning between screens, etc.) is required to complete this task	○	○	○	○	○	○
I have to work very hard (mentally and physically) to perform this task	○	○	○	○	○	○
I feel very irritated, stressed, or annoyed during this task	○	○	○	○	○	○
This task feels unnecessary	○	○	○	○	○	○
This task feels unreasonable	○	○	○	○	○	○
This task is not relevant to improving patient care or outcomes	○	○	○	○	○	○

Applied Clinical Informatics | DOI 10.1055/a-2751-1896 | © The Author(s).

Task Evaluation For each statement, indicate your level of agreement by selecting the option that best reflects your opinion

Task: Billing/Coding

	Very Strongly Disagree	Strongly Disagree	Disagree	Agree	Strongly Agree	Very Strongly Agree
Performing this task is unnecessarily complex	○	○	○	○	○	○
Performing this task is easy to do	○	○	○	○	○	○
The necessary functions to complete this task are well-integrated	○	○	○	○	○	○
There is too much inconsistency when trying to complete this task	○	○	○	○	○	○
An excessive amount of mental activity (concentration, recall, etc.) is required to perform this task	○	○	○	○	○	○
An excessive amount of physical activity (clicking, scrolling, transitioning between screens, etc.) is required to complete this task	○	○	○	○	○	○
I have to work very hard (mentally and physically) to perform this task	○	○	○	○	○	○
This task feels unnecessary	○	○	○	○	○	○
This task feels unreasonable	○	○	○	○	○	○
This task is not relevant to improving patient care or outcomes	○	○	○	○	○	○

Applied Clinical Informatics | DOI 10.1055/a-2751-1896 | © The Author(s).

Feelings about Burden This section asks you to share your thoughts on clinical documentation burden

Does the burden of clinical documentation create a conflict between your personal values as a caregiver and the values of the healthcare system? If yes, how? ________________________________

Do clinical documentation demands impact your emotional well-being at work? If yes, how? ________________________________

Is there anything that was not asked about in this survey on documentation burden that you think we should have included? ________________________________

Applied Clinical Informatics | DOI 10.1055/a-2751-1896 | © The Author(s).

About You This section asks some basic demographic questions so we can learn a little more about you

What is your age?	◯ 18-25<br>◯ 25-34<br>◯ 35-44<br>◯ 45-54<br>◯ 55-64<br>◯ 65 and over
What term best describes your gender?	◯ Male<br>◯ Female<br>◯ Non-binary/third gender<br>◯ Prefer to self-describe ______<br>◯ Prefer not to say
What is your current role?	◯ Resident<br>◯ Physician<br>◯ Advanced Practice Nurse (e.g. APRN, CNS, CRNA)<br>◯ Physician Assistant<br>◯ Other ______
What is your primary specialty?	◯ Primary Care/Generalist (i.e. Family Medicine, Geriatrics)<br>◯ Medical Specialty (i.e. Cardiology, Endocrinology)<br>◯ Surgical Specialty (i.e. Orthopedic Surgery, Plastic Surgery)<br>◯ Other Specialty (i.e. Psychiatry, Obstetrics & Gynecology) ______
How many years of experience do you have as a healthcare provider?	◯ Less than 5 years<br>◯ 6-10 years<br>◯ 11-15 years<br>◯ 16-20 years<br>◯ More than 20 years
What is the highest degree you've achieved?	◯ Master's degree<br>◯ Doctorate or Professional degree
On average, how many hours do you work per week (include all clinical and non-clinical duties)?	◯ Less than 20<br>◯ 21-30<br>◯ 31-40<br>◯ 41-50<br>◯ 51-60<br>◯ 61-70<br>◯ 71-80<br>◯ More than 81
How would you describe your primary practice setting?	◯ Hospitals<br>◯ Ambulatory Care Settings<br>◯ Long-term Care Facilities<br>◯ Home Health Care<br>◯ Rehabilitation Centers<br>◯ Community Health Centers<br>◯ Urgent Care Centers<br>◯ Private or Group Practice<br>◯ Other ______
Does your practice setting have an academic affiliation?	◯ Yes<br>◯ No

Applied Clinical Informatics | DOI 10.1055/a-2751-1896 | © The Author(s).

Appendix B. Constructs, Variables & Operational Definitions related to Clinical Documentation Burden

Constructs / Variables	*Definition*	*Response Scale*
Documentation Activity Task	Clinically meaningful units of work organized into the activities reflect core clinical, communication, and clerical functions of providers, accounting for 95% of their time in the EHR	Categorical – Derived from prior study (Arndt et al, 2017)
Time spent on activity/tasks	Self-reported average minutes the clinician spends on a specific EHR documentation activity task during a typical shift/day	Continuous - Open numeric (0.5 hour increments)
Perceived overall burden	Respondent asked to order all seven EHR documentation tasks according to perceived burden level	Ordinal – Forced global ranking from highest (1) to lowest (7)
**Usability**	User's overall judgment of how efficiently, effectively, and satisfactorily the system enables the completion of required tasks	Items Derived from System Usability Scale (Brooke, 1996)
System Complexity	The perception that the system's structure, navigation paths, or required actions are overly intricate	Six-point Likert: Very strongly disagree (1) to Very strongly agree (6)
Perceived Ease of Use	Perceived effort of interaction—the extent to which the user feels that completing core task is intuitive and straightforward	Six-point Likert: Very strongly disagree (1) to Very strongly agree (6)
Functionality Integration	User's perception that the system's individual features form a coherent, smoothly coordinated whole	Six-point Likert: Very strongly disagree (1) to Very strongly agree (6)
System Inconsistency	Perceived lack of uniformity in commands, layout, terminology, or behavior across the system	Six-point Likert: Very strongly disagree (1) to Very strongly agree (6)
**Effort**		Items derived from NASA Task Load Index (Hart, 2006)

Excess mental activity	Asks about the amount of cognitive activity (e.g. thinking, deciding, remembering) the task requires	Six-point Likert: Very strongly disagree (1) to Very strongly agree (6)
Excess physical activity	The amount of physical or psychomotor effort needed (e.g., clicking, scrolling).	Six-point Likert: Very strongly disagree (1) to Very strongly agree (6)
Effort Required	How hard the user had to work (mentally and physically) to accomplish the task	Six-point Likert: Very strongly disagree (1) to Very strongly agree (6)
Emotional Strain	Focuses on how insecure, discouraged, irritated, or stressed the user felt during use	Six-point Likert: Very strongly disagree (1) to Very strongly agree (6)
**Value**	Considers the dynamic between professional values, task alignment and the impact on intrinsic motivation and perceived burden	Items derived from Bern Illegitimate Tasks scale (Semmer et al., 2010)
Necessity	Does the task feel pointless or avoidable (e.g., duplicate data entry	Six-point Likert: Very strongly disagree (1) to Very strongly agree (6)
Reasonableness	Does the participant believe the task should rightfully belong to someone else (e.g., basic data entry by a physician)	Six-point Likert: Very strongly disagree (1) to Very strongly agree (6)
Relevance	The relevance of a task, i.e. whether it directly informs clinical decision making or contributes to safe longitudinal care, modulates its perceived burden to a clinician	Item derived from findings in article on "Excessive DocBurden" (Levy et al., 2024)<br>Six-point Likert: Very strongly disagree (1) to Very strongly agree (6)
**Professional Dissonance**	The tension that arises when the tasks, behaviors, or values demanded by a work context conflict with the norms of a competent, ethical member of a profession (e.g. the documentation providers are required to complete for reimbursement distracts from their clinical purpose)	Items derived from Bern Illegitimate Tasks scale serve as proxy<br>Narrative-prompt item embedded in the survey, coded dichotomously
**Burnout**	Work-related syndrome marked by emotional exhaustion, depersonalization, and a diminished sense of professional efficacy or accomplishment.	Narrative-prompt item embedded in the survey, coded dichotomously

Appendix C. Full Factor Loading Matrix

Matrix Name	#	Factor Description	Q1 complex	Q2R not easy	Q3R poor integration	Q4 inconsistent	Q5 mental load	Q6 physical load	Q7 work hard	Q8 irritated	Q9 unnecessary	Q10 unreasonable	Q11 not relevant
Chart Review	1	consistent poor usability, irritating task load/burden	**0.774**	***-0.674***	***-0.662***	0.442	**0.736**	**0.711**	**0.756**	**0.76**	-0.169	0.289	-0.086
Chart Review	2	illegitimate and not relevant	0.011	-0.175	0.152	0.156	-0.048	-0.116	-0.062	0.015	**.959**	**0.575**	**0.826**
Writing Notes	1	poor usability	**0.526**	**-.609**	***-0.886***	**0.582**	-0.108	0.371	0.073	0.243	-0.052	0.131	-0.04
Writing Notes	2	illegitimate and not relevant	0.098	0.04	0.08	0.067	-0.013	0.026	-0.015	0.16	**0.989**	**0.862**	**0.783**
Writing Notes	3	irritating cog load burden	0.063	-0.254	0.043	-0.072	**0.84**	0.327	**0.754**	**0.676**	-0.029	0.046	-0.002
Problems/Dx	1	not irritating task load burden	0.215	-0.449	-0.138	0.011	**0.869**	**0.76**	**0.836**	0.355	-0.108	-0.001	0.1
Problems/Dx	2	illegitimate and not relevant	0.082	-0.164	-0.186	-0.091	-0.02	0.07	-0.097	0.08	**0.857**	**0.675**	**0.87**
Problems/Dx	3	simple and consistent task	***-0.653***	0.34	0.38	***-0.593***	-0.085	-0.037	0.06	-0.47	-0.152	-0.4	0.338
Order Entry	1	inconsistent poor usability, irritating task load/burden	**0.772**	***-0.671***	***-0.715***	**0.514**	**0.754**	**0.563**	**0.761**	**0.731**	0.066	0.168	-0.062
Order Entry	2	illegitimate and not relevant	-0.014	-0.108	-0.164	0.171	-0.192	0.135	-0.354	0.062	**0.905**	**0.898**	**0.905**
Billing/Coding	1	consistent poor usability, cog load	**0.641**	***-0.908***	***-0.634***	0.469	**0.762**	0.415	0.471		0.051	0.124	-0.112
Billing/Coding	2	illegitimate and not relevant	0.256	0.179	-0.022	0.433	0.088	-0.053	0.078		**0.856**	**0.808**	**0.858**
Administrative	1	inconsistent poor usability, non-irritating task load/burden	**0.709**	***-0.78***	***-0.645***	**0.693**	**0.826**	**0.681**	**0.669**	**0.496**	0.004	0.171	-0.045
Administrative	2	illegitimate and not relevant	0.127	-0.177	-0.195	0.188	-0.169	-0.098	-0.203	0.219	**0.979**	**0.781**	**0.717**
